# Medical Fashions

**Published:** 1920-07-03

**Authors:** 


### MEDICAL FASHIONS.

In the course of a recent letter to the *Glasgow Herald*, a correspondent who was casting doubt upon the value of vaccination, said: "Medical fashions come and go." We are not concerned to labour the question of vaccination. That has been settled in every mind open to conviction, but the phrase suggests other thoughts.

The writer of the letter doubtless intended to infer that medical opinion is fickle and unstable, teaching one thing to-day and another to-morrow. Making allowance for the inevitable exaggerations of general statements, we admit the soft impeachment, not only with equanimity, but also with enthusiasm. Medicine is an empirical and not yet an exact science; but it is progressive, and progress based upon empiricism is, of necessity, responsible for frequent changes of view, and the gradual discarding of old teaching in so far as this is shown to be imperfect by increasing light and knowledge.

Our claim is, however, that each successive change is an improvement upon its predecessor, change of front being the result of ascertained and proved facts. Medical opinion is not merely arbitrary. It may be imperfect, but it is not unreasonable. And, unless further progress is to be denied to medicine, the best we know at any given time must be allowed full scope in demonstration and application.

We must confess, however, that there have been instances in which opinion has swung so rapidly from one extreme to the other as to give the enemy cause to blaspheme. Two instances must suffice. In the matter of drugs the fashion has indeed changed Not so long ago charges of small shot in the shape of a dozen or more drugs in a bottle, were hurled at the unfortunate patient in the hope that one, at any rate, might find its billet. This was ridiculous from any point of view. But nowadays we hear constantly of medical men boasting that they "never give medicines." This is no less absurd. There is unreason in both attitudes. The fact that advancing knowledge indicates in the future of medicine "a more excellent way" than our present imperfect methods of medication is no reason for allowing a child to lie awake coughing when a simple linctus would produce comfort, and thereby induce sleep with all its beneficent results. Yet this, in the absence of the writer, was the attitude adopted towards his child by a London M.D. and hospital lecturer, who "did not believe in medicine."

What was the result? What often *is* the result in cases such as this? Patent cough-mixtures from the chemist! The doctor must use with discretion the remedies he has, and not leave his patients in the lurch through anticipating events that have not yet materialised. The late Editor of this Journal once said to the writer: "The G.P.'s job is to make people comfortable."

One other example of violent, indiscriminating change of opinion, dangerous and altogether unwarrantable. In the matter of infection in tuberculosis, the teaching until recently laid every possible stress upon the dangers of contact, etc. Modern research is gradually discovering more fully the life history of the bacillus, and is in process of modifying previous opinion in the matter of infection. The obvious duty of the medical profession is to exercise all discrimination and care in its teaching until the subject has been thoroughly elucidated. Unfortunately we find even some tuberculosis experts speaking so loosely as to give the public the impression that all are infected, and that the only bacilli to be feared are those implanted in each individual. This is a swing of the pendulum from one extreme to the other, a change of front fraught with the gravest dangers. Change of opinion should rightly keep pace with increase of knowledge, but it should not anticipate it.

"Prove all things; hold fast that which is good."

